# Results of a clinical practice algorithm for the management of thoracostomy tubes placed for traumatic mechanism

**DOI:** 10.1186/2193-1801-2-642

**Published:** 2013-12-01

**Authors:** Mersadies Martin, Cory T Schall, Cheryl Anderson, Nicole Kopari, Alan T Davis, Penny Stevens, Pam Haan, John P Kepros, Benjamin D Mosher

**Affiliations:** Department of Surgery, Michigan State University College of Human Medicine, 1200 E Michigan Ave, Suite 655, Lansing, MI 48912 USA; Michigan State University College of Human Medicine, 965 Fee Rd., Room A-110, East Lansing, MI 48824 USA; Grand Rapids Medical Education Partners, Department of Surgery, Michigan State University College of Human Medicine, 1000 Monroe NW, Grand Rapids, MI 49503 USA; Sparrow Health System, 1215 E. Michigan Ave, 2 South, Trauma Services, Lansing, MI 48912 USA

**Keywords:** Tube thoracostomy, Algorithm, Guideline, Pneumothorax, Hemothorax, Outcomes

## Abstract

**Introduction:**

The management and removal of thoracostomy tubes for trauma-related hemothorax and pneumothorax is controversial. General recommendations exist; however, institutional data related to an algorithmic approach has not been well described. The difficulty in establishing an algorithm centers about individualized patients’ needs for subsequent management after thoracostomy tube placement. In our institution, we use the same protocol for all trauma patients who receive a thoracostomy tube with minimal complications.

**Purpose:**

To present the clinical outcomes of patients who required a tube thoracostomy for traumatic injury and were managed by an institutional protocol.

**Methods:**

A retrospective chart review of 313 trauma patients at a single level I trauma institution from January 2008 through June 2012 was conducted. Inclusion criteria were patient age ≥ 18 years, involvement in a trauma, and requirement of a thoracostomy tube. The patients’ charts were reviewed for demographic data, injury severity score (ISS), length of stay (LOS), and chest-tube specific data. Thoracostomy tube complications were defined as persistent air leak, persistent pneumothorax, recurrent pneumothorax, and clotting of thoracostomy tube. The patients were managed per our institutional algorithm. Descriptive statistics were performed.

**Results:**

Most of the patients who required a thoracostomy tube had blunt-related traumas (271/313; 86.6%), while 42 patients (13.4%) sustained penetrating injuries. There were 215 (68.7%) male patients. The average age at time of injury was 45.7 ± 21.1 years and the mean ISS was 24.9 ± 15.9 (mean ± SD). Elevated alcohol levels were found in 65 of the 247 patients who were tested upon admission (26.3%). Overall, 15 patients (4.8%) developed a thoracostomy tube related complication: persistent air leak in six patients, persistent pneumothorax in six patients, recurrent pneumothorax in two patients, and clotted thoracostomy tube in one patient. The average LOS was 10.4 ± 8.4 days, and the mean length of thoracostomy tube placement was 5.9 ± 4.3 days.

**Conclusions:**

Our algorithmic thoracostomy tube management protocol resulted in a complication rate of 4.8%. By managing thoracostomy tubes in a systematic manner, our patients have improved outcomes following placement and removal compared to other studies.

## Background

Much controversy exists regarding the management of tube thoracostomy, or chest tube placement, in the postoperative period and only a few algorithms exist in the management of thoracostomy tubes for the treatment of pneumothorax (PTX), hemopneumothorax, hemothorax, flail chest, and tension PTX following blunt or penetrating traumatic injury. General recommendations exist; however, institutional data related to an algorithmic approach in trauma patients is not well described. The ideal thoracostomy tube management algorithm has yet to be determined. The difficulty in establishing an algorithm centers about individualized patients’ needs for subsequent management after thoracostomy tube placement. In our institution, we use the same protocol for all trauma patients who receive a thoracostomy tube despite their individualized needs.

The objective of this study was to retrospectively analyze the clinical outcomes and complication rates of our thoracostomy tube management protocol and then review and compare the literature with the possibility of implementing our algorithm as a general guideline for all institutions, and more importantly improve patient care. The complication rates, duration of thoracostomy tubes and length of hospital stay were collected as primary outcome variables. Secondary outcome variables included the injury severity scores in relation to thoracostomy tube duration, mechanism of trauma, time of air leak, suction, water seal, and the relative thoracostomy tube outputs in relation to complication rates.

## Results

There were 313 patient records reviewed for the study. Demographic and clinical data are described in Table 1. Most of the patients were male, and sustained a blunt injury. Alcohol was involved in 65 of the 247 patients that were tested (26.3%). Fifteen subjects sustained a complication. Patients without a complication had an ISS of 24.6 ± 15.9, while the 15 patients who sustained a complication had an injury severity score (ISS) of 30.7 ± 18.3 (p = 0.22).

**Table 1:** **Demographics**

	Demographics
Age (years)	45.7 ± 21.1
Gender	
Male	215/313 (68.7%)
Female	98/313 (31.3%)
Mechanism of Injury	
Blunt	271/313 (86.6%)
Penetrating	42/313 (13.4%)
Injury Severity Score (mean)	24.9 ± 15.9
Length of Stay (mean days)	10.4 ± 8.4
Chest Tube time (mean days)	5.9 ± 4.3

Table 2 lists the individual complications with their absolute numbers. Of the reported complications, two patients underwent a thoracotomy, nine patients required an additional thoracostomy tube, two patients required a thoracostomy tube to be replaced with a larger thoracostomy tube, and two patients were managed with continued suction until resolution of PTX. The most common complications were persistent air leak and persistent PTX, which were each documented in six patients.

**Table 2:** **Complication rates**

Complication rates of tube thoracostomy	Number (%)
Persistent air leak	6 (4)
Persistent Pneumothorax	6 (4)
Recurrent Pneumothorax	2 (1.3)
Post placement infection	0
Non-functional: clotted	1 (0.7)
Non-functional: positional	0
Non-functional: kinked	0
Sum of Complications	15 (4.8)

## Discussion

The objective of this study was to evaluate our thoracostomy tube management protocol following a trauma-induced event. We decided to retrospectively collect data among all the trauma patients who we have consistently used this algorithm on in hopes to not only improve patient care but also to share the algorithm due to its simplicity and effectiveness. In our practice, the mechanism of injury does not alter the management of tube thoracostomy, however, complication rates may vary depending on the severity of injury.

In our analysis, 15 of the 313 patients (4.8%) were found to have a thoracostomy tube complication. This was low compared to other studies. Menger et al. conducted a retrospective chart review in 154 patients with a 22.1% thoracostomy tube complication rate following thoracic trauma (Menger et al. 2012). They concluded that the severity of injury (measured by the abbreviated injury score) should be incorporated into the development of thoracostomy tube management guidelines. Patients in our study had a numerically higher ISS score than patients without complication, although this difference did not achieve statistical significance.

In 1995, Etoch et al. conducted a retrospective institutional review and displayed a 21% complication rate associated with thoracostomy tube management in 379 trauma patients (Etoch et al. 1995). However, the primary outcome of this study was a comparison regarding the complication rate after insertion by a surgeon (6%), an emergency medicine physician (13%), or prior to the transfer of the patient (38%).

In 2000, a retrospective case series determining the complication of tube thoracostomy in trauma patients was 30%. The objective was to determine if the rate was high enough to support a selective reduction in the indications for tube thoracostomy. The conclusion of the study revealed no persuasive evidence to support a selective reduction and the need for a larger study to confirm or refute their findings (Bailey 2000). In 1997, Chan et al. and Collop et al. reported complication rates of 11% and 18.2%, respectively (Chan et al. 1997; Collop et al. 1997). An algorithm regarding thoracostomy tube management with the above portrayed complication rates were not described in any of the above studies.

Based upon the available medical literature and clinical expertise, the Department of Surgical Education at the Orlando Regional Medical Center presented an algorithm similar to what we use in our institution (Cheatham 2009). However, this was based upon literature from other studies rather than upon results from their institution. It was not published as a peer-reviewed document, nor was it used in patients who were not involved in a trauma. In this evaluation, they depicted data based on a defined level of published data. Their recommendations regarding level-one evidence suggested that thoracostomy tube drainage should be ≤ 2 mL/kg/day or ≤ 200 mL/day before removal. Our algorithm suggests that thoracostomy tube output should be ≤ 200 mL/day to advance from wall suction to water seal or water seal to removal. These data were based on randomized studies evaluating the timing of thoracostomy tube removal in regards to the daily drainage volume (Younes et al. 2002; Hessami et al. 2009). Thus, both Hessami et al. and Younes et al. conducted prospective randomized investigations of 138 and 139 trauma patients and documented that a thoracostomy tube output of < 200 mL/day was just as safe as an output of <150 mL/day.

In regards to placing a thoracostomy tube on continuous suction after insertion, Davis and colleagues randomized 80 patients to wall suction versus water seal and observed a similar incidence of recurrent PTX (2.5%) in both groups (Davis et al. 1994). However, they concluded that the suction algorithm could help reduce the length of stay by reducing the total thoracostomy tube time (72.2 hours versus 92.5 hours, P = 0.013), as well as removal time (25.2 hours versus 35.6 hours, P = 0.034).

Interestingly, Martino et al. published a prospective randomized study in 205 trauma patients in which their thoracostomy tubes were removed either on water seal or on wall suction (Martino et al. 1999). Following thoracostomy tube removal, a recurrent PTX was seen in 13 patients in the water seal group with only one patient requiring tube replacement and a recurrent PTX in nine patients from the suction group with seven patients requiring a tube replacement. The study concluded that the water seal group was more likely to have recurrent PTX after thoracostomy tube removal but less likely to need a replacement.

Per our algorithm, we do not remove thoracostomy tubes on wall suction. Martino et al. concluded that a trial of water seal appears to allow occult air leaks to become clinically apparent, thus potentially reducing the need for another thoracostomy tube (Martino et al. 1999). Furthermore, regarding wall suction versus water seal, the literature has suggested that suction compared to water seal does not reduce air leaks in patients whom have had a pulmonary resection. However, it could decrease the occurrence of postoperative PTX from early air leak (Deng et al. 2010). Further, this study did not involve trauma patients. We do not have data to compare the recurrence of PTX after a thoracostomy tube is removed on water seal versus wall suction, which is a limitation of our study.

Schulman et al. prospectively evaluated the time interval for identifying a PTX after placing 119 thoracostomy tubes on water seal for three hours then obtaining a chest x-ray (CXR) (Schulman et al. 2005). They concluded that 31 patients had a PTX on follow-up CXR, 22 were identified early and nine were late. Of the 22 patients identified early, three had a clinically significant increase in size of a PTX. This may suggest that three hours of water seal time may not be safe. Our algorithm is based on approximately 24 hours of water seal time before removal with only 2/313 patients (1.3%) having a recurrent PTX.

In regards to removing a thoracostomy tube, we obtain a thoracostomy tube CXR ≥ 4 hours after removal. From our data, two of the patients (1.3%) developed a recurrent PTX after removal and these patients were observed without an intervention. Bell et al. revealed that up to 24% of patients might have a small apical PTX after thoracostomy tube removal that does not require a repeat thoracostomy tube (Bell et al. 2001). Interestingly, this prospective randomized study also compared the removal of 102 thoracostomy tubes in 69 trauma patients, either at the end of inspiration or at the end of expiration, and found no significant difference in the recurrence of a PTX. In 2000, Pacanowski et al. conducted a retrospective review of 105 patients with 113 thoracostomy tubes removed with a protocol CXR performed eight to 22 hours after removal (Pacanowski et al. 2000). The authors advocated obtaining a CXR 24 hours after thoracostomy tube removal.

The median chest tube drainage time for patients in the study of Younes et al. was approximately three days (Younes et al. 2002). However, there were three treatment arms in this study, and chest tubes were withdrawn depending on the amount of pleural fluid that was drained (<200 mL/day, <150 mL/day, and <100 mL/day), whereas in our study all patients were removed from suction once their drainage was below 200 mL/day. Even though we had an average drainage time of 5.9 days, it is likely that the 313 patients in our study had experienced more serious injuries than the 37 patients who were in the <200-mL/day-drainage group in the study of Younes et al., which would explain the increased chest tube drainage time.

The mean length of hospital stay in our study was 10.4 days. Although Hessami et al. reported a mean hospital stay of 4.1 days, it is possible that this difference can be attributed to more severely injured patients in our study who required longer hospital care (Hessami et al. 2009). Hessami had 138 patients who required chest tube placement due to malignancy and trauma, while all of our patients had trauma injuries, which could contribute to longer hospital duration.

The policy in our department with regard to thoracostomy tube size is in line with the Advanced Trauma Life Support^®^ (ATLS) recommendations, such that a 32–40 French (F) drain should be used for a trauma-induced PTX or hemothorax. Collop et al. found a 36% complication rate associated with a tube thoracostomy of 14 F or less compared to a 9% rate for a standard tube (Collop et al. 1997). In general, the use of a large bore 32 F or greater is likely to reduce the complication associated with a drain becoming kinked or clotted. In our study, one patient with a 32 F thoracostomy tube clotted, requiring a thoracostomy and replacement with two 32 F thoracostomy tubes. Interestingly, Inaba et al. conducted a prospective analysis and compared the efficacy of small (28–32 F) versus large (36–40 F) thoracostomy tubes in 293 patients with thoracic traumas and concluded no differences in retained hemothoraces, the need for additional tube insertion and/or pain level (Inaba et al. 2012).

Occult PTX is a controversial situation that may arise in rare circumstances. By definition, an occult PTX is a PTX identified by computed tomography (CT) scan but not by CXR. We have included information in our algorithm as to how we manage occult pneumothoraces. However, a large prospective randomized study is needed in trauma patients to guide the management. We did not evaluate the number of patients who were admitted with a trauma-induced occult PTX that did not require a thoracostomy tube, which was a limitation of this study.

## Conclusions

Overall, we believe in practicing safe precautions. The development of just one complication requires careful surveillance. Based on the presented evidence and review of literature, we believe thoracostomy tubes may be removed safely if no air leak is present, drainage is < 200 mL/day, and if a water-sealed CXR revealing a stable or improved PTX <10% is obtained before thoracostomy removal. Following the removal of a thoracostomy tube, we believe a CXR should be obtained. In conclusion, our algorithm is an institutional evidence-based example implying a simplistic guidance tool that may be used at other institutions with the potential of becoming an accepted protocol. Although there are areas needing further study, we believe that a prospective randomized study at a different institution using our protocol would be beneficial to confirm or refute the use of our algorithm.

## Materials and methods

**Figure 1:**
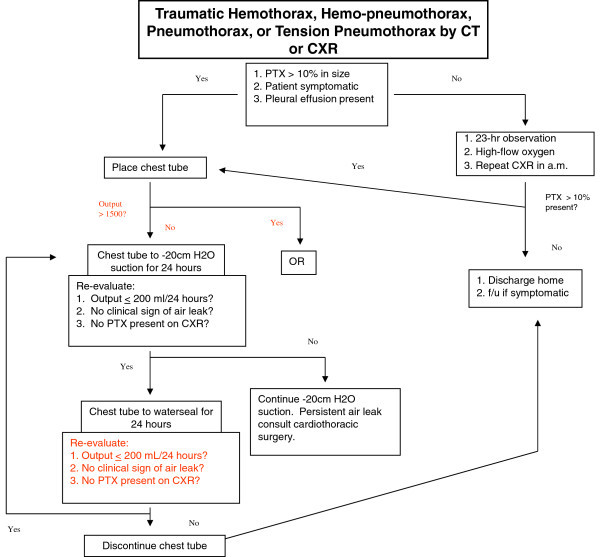
**Thoracostomy tube management algorithm.**

A retrospective analysis of all trauma patients who underwent tube thoracostomy for PTX, hemothorax, hemopneumothorax, flail chest, or tension PTX between the time period of January 2008 to June 2012 at Sparrow Hospital, Lansing, Michigan. Institutional Review Board approval was received. The management of each patient was essentially the same using an algorithmic approach presented in Figure 1. These patients were identified by the trauma audit department computerized retrieval system and sorted for the tube thoracostomy or chest tube insertion procedure code. Further analysis and data retrieval was conducted via hospital chart review. Exclusion criteria included patients who required a thoracostomy tube that were not involved in a trauma, patients who expired before the removal of thoracostomy tube that was not related to thoracostomy management, and patients who required a cardiothoracic consultation within 24 hours of admission. Patients that required an urgent cardiothoracic consultation included an initial thoracostomy output >1500 mL, more than 200 mL/hour output for 3 hours, or an obvious thoracic chest injury that required surgery.

Retrospective analysis included information such as age, sex, mechanism of injury, indication(s) for thoracostomy placement, duration of thoracostomy tube insertion, length of hospital stay, ISS, thoracostomy tube specific data (i.e., duration of air leak, water seal, suction time, thoracostomy tube output, size of thoracostomy tube), and complication data. Complications were defined as persistent leak, persistent or recurrent pneumo- or hemothoraces, post placement infection, and non-functional thoracostomy tube (i.e., clotted, positional, kinked).

All thoracostomy tubes were inserted using the open blunt dissection technique recommended by the British Thoracic Society and the American College of Surgeon guidelines (Laws et al. 2003; ACS Committee on Trauma 2013). Indications for thoracostomy tube insertion consisted of a trauma-related PTX >10%, hemothorax, hemopneumothorax, flail chest, and tension PTX.

All thoracostomy tubes were managed following our institutional algorithm (Figure 1) as described in this paragraph. If a thoracostomy tube was not required, the patients were managed overnight with a repeat CXR the following morning. If the repeat CXR was stable, the patient was discharged the same day. If a thoracostomy tube was required, it was immediately placed to a wall-mounted vacuum (i.e., ‘wall suction’) at -20 cm H_2_O and a post-thoracostomy tube placement CXR was obtained. The following morning a repeat CXR was obtained and the thoracostomy tube was placed on water seal if the output was < 200 mL, there was no air leak, and if a repeated CXR revealed no PTX or a stable PTX that was <10% in size. If those conditions were not met, the thoracostomy tube was kept on suction until a CXR was repeated the next day. If the patient required continuous suction for more than 3 days, a cardiothoracic consultation was required for persistent air leak or PTX. On the contrary, the thoracostomy tube could be removed if upon re-evaluation the following day (after being on water seal) reported output as < 200 mL, no air leak, and if the repeated CXR revealed no PTX or a stable PTX that was <10% in size. Approximately 4 hours after the thoracostomy tube was removed a CXR was obtained. Depending on the results of the CXR, the patient went home the same day, was observed overnight with a repeat CXR the following day, or had a second thoracostomy tube placed. If there was a persistent air leak, persistent or recurrent pneumo- or hemothorax, or continual thoracostomy tube drainage, a CT scan of the chest was obtained. Depending on the results, a second tube was placed versus a cardiothoracic consultation (for possible video-assisted thoracoscopy, lobectomy, or pleurodesis).

Summary statistics were calculated for the data. Quantitative variables are expressed as the mean ± SD, while nominal variables are expressed as a percentage. ISS scores were compared between patients with and without complications using the two-tailed *t*-test. Significance was assessed at p < 0.05.

## Abbreviations

ISS: Injury severity score
PTX: Pneumothorax
CXR: Chest x-ray
ATLS: Advanced Trauma Life Support^®^
CT: Computed tomography.
